# Bioactivity, Molecular Mechanism, and Targeted Delivery of Flavonoids for Bone Loss

**DOI:** 10.3390/nu15040919

**Published:** 2023-02-12

**Authors:** Ashish Ranjan Sharma, Yeon-Hee Lee, Altanzul Bat-Ulzii, Srijan Chatterjee, Manojit Bhattacharya, Chiranjib Chakraborty, Sang-Soo Lee

**Affiliations:** 1 Institute for Skeletal Aging & Orthopedic Surgery, Hallym University-Chuncheon Sacred Heart Hospital, Chuncheon-si 24252, Gangwon-do, Republic of Korea; 2 Department of Zoology, Fakir Mohan University, Vyasa Vihar, Balasore 756020, Odisha, India; 3 Department of Biotechnology, School of Life Science and Biotechnology, Adamas University, Kolkata 700126, West Bengal, India

**Keywords:** flavonoids, signaling mechanism, delivery methods, therapeutics, bone loss

## Abstract

Skeletal disabilities are a prominent burden on the present population with an increasing life span. Advances in osteopathy have provided various medical support for bone-related diseases, including pharmacological and prosthesis interventions. However, therapeutics and post-surgery complications are often reported due to side effects associated with modern-day therapies. Thus, therapies utilizing natural means with fewer toxic or other side effects are the key to acceptable interventions. Flavonoids constitute a class of bioactive compounds found in dietary supplements, and their pharmacological attributes have been well appreciated. Recently, flavonoids’ role is gaining renowned interest for its effect on bone remodeling. A wide range of flavonoids has been found to play a pivotal role in the major bone signaling pathways, such as wingless-related integration site (Wnt)/β-catenin, bone morphogenetic protein (BMP)/transforming growth factor (TGF)-β, mitogen-activated protein kinase (MAPK), etc. However, the reduced bioavailability and the absorption of flavonoids are the major limitations inhibiting their use against bone-related complications. Recent utilization of nanotechnological approaches and other delivery methods (biomaterial scaffolds, micelles) to target and control release can enhance the absorption and bioavailability of flavonoids. Thus, we have tried to recapitulate the understanding of the role of flavonoids in regulating signaling mechanisms affecting bone remodeling and various delivery methods utilized to enhance their therapeutical potential in treating bone loss.

## 1. Introduction

Skeletal disabilities are a prominent modern-day problem and have become a concern lately. An increase in life expectancy and a growth in the elderly population worldwide have substantially burdened the existing health systems [1,2]. Bone is a living organ, constituting 30% organic and 70% inorganic material with various functions such as protecting internal organs, making the body frame, and safe storage for some vital minerals in the body [3,4]. There are various classes of bone cells, such as osteoblasts, osteocytes, bone-lining cells, and osteoclasts [4]. All these cells are responsible for bone metabolism, characterized by a constant equilibrium of bone formation (by the osteoblasts) and bone resorption (mediated by osteoclasts). Despite that, the disruption between bone formation and bone resorption contributes to several metabolic bone disorders, namely osteoporosis, osteopetrosis, and Paget’s disease [5,6]. Osteoporosis is regarded as a health problem affecting over 200 million people globally, according to the World Health Organization [7]. The crucial risk factor for osteoporosis is age-associated bone loss, which occurs in people over 50 years of age, including approximately 25% of men and 50% of women [8]. Osteoporosis can be categorized into two main groups, primary and secondary. Primary osteoporosis is the most typical type and is mainly caused by loss of bone mass during the aging process or in postmenopausal women because of decreased estrogen levels. Secondary osteoporosis is associated with lifestyle, secondary systemic disorders such as diabetes, hypothyroidism, etc., and long-term use of drugs such as glucocorticoids [9]. The method of treating bone loss during osteoporosis is to stimulate bone growth. The understanding of why a difference in normal osteoblastogenesis regulation can result in bone disorders has improved due to recent advancements and expanded knowledge in the regulation of osteoblastic bone growth and maintenance of bone mass [10].

The current therapeutic approach to treat bone loss includes either inhibiting elevated bone resorption or stimulating suppressed bone formation. Drugs used for antiresorptive characteristics included bisphosphonates, hormone replacement therapy (HRT) (estrogen), Raloxifene, and monoclonal such as Denosumab and Romosozumab; while for inducing bone formation and increasing bone density, anabolic drugs such as Teriparatide (parathyroid hormone/PTH 1-34) are recommended [11]. However, estrogen replacement therapy has been found to be associated with heart attack, stroke, and risk of cancer. The use of Teriparatide as a drug for osteoporosis has highlighted the risk of osteosarcoma in rodent models [12]. Moreover, long-term bisphosphonates treatment could lead to skeletal lesions, developing into bisphosphonate-related osteonecrosis of the jaw [11,13]. Though the commercially available drugs have been remarkable in treating bone loss during osteoporosis, a few cases of side effects may make them consider before prescribing. Hence, finding a new, effective, safe therapeutic agent with no or fewer side effects is essential for bone loss pathologies.

Flavonoids are present in dietary supplements, including vegetables, grains, fruits, stems, bark, flowers, etc. [11,14] and have been well acknowledged for their diverse bioactivities, including anti-oxidant, anti-allergic, anti-inflammatory, anti-carcinogenic, and antiviral activities [15]. The flavonoids contain over 5000 polyphenol compounds and are divided into seven flavonoid groups: anthocyanidins, flavanols, flavanones, flavanonols, flavones, flavonols, and isoflavones, categorized on the presence of hydroxyl groups structure and their glycosylation or alkylation status [16,17]. Recent studies on flavonoids have shown their notable effect on bone cells, such as increasing osteoblast activity, suppressing osteoclast activity, and protecting against bone loss, in addition to decreasing calcium and phosphate urinary excretion [18]. Some in vivo studies proved that flavonoids, such as Daidzein, Quercetin, Kaempferol, and Genistein, could affect osteogenesis and bone formation, while some studies report inhibiting effect on osteoclastogenesis and bone resorption [19,20]. Some of the flavonoids, such as isoflavones, accelerate bone formation by inducing osteoblasts differentiation and cell proliferation along with the inhibition of adipogenesis through the nitric oxide and estrogen receptor pathways [21,22]. 

Low systemic bioavailability is a general problem for flavonoids [23]. Mostly, it is associated with the absorption of flavonoids. The majority of flavonoids reach the colon unabsorbed [24]. Even then, several studies have reported the efficacy of flavonoids for bone health, such as stimulating osteoblastogenesis in in vitro and in vivo models [25,26,27,28]. Overcoming the low absorption and bioavailability of flavonoids, recent advancements in delivery systems are offering flavonoids as potential therapeutics for bone loss. Delivery vehicles such as nanoparticles, micelles, biomaterials, and scaffolds are commonly used carriers of flavonoids for bone [14,29]. In this review, we summarize the potential effect of flavonoids on stimulating bone formation by regulating various signaling pathways. In addition, the variety of methods used for delivering the flavonoids is also discussed to assess their possibility as a next-generation therapeutic for bone loss.

## 2. Types of Flavonoids

**Figure 1:**
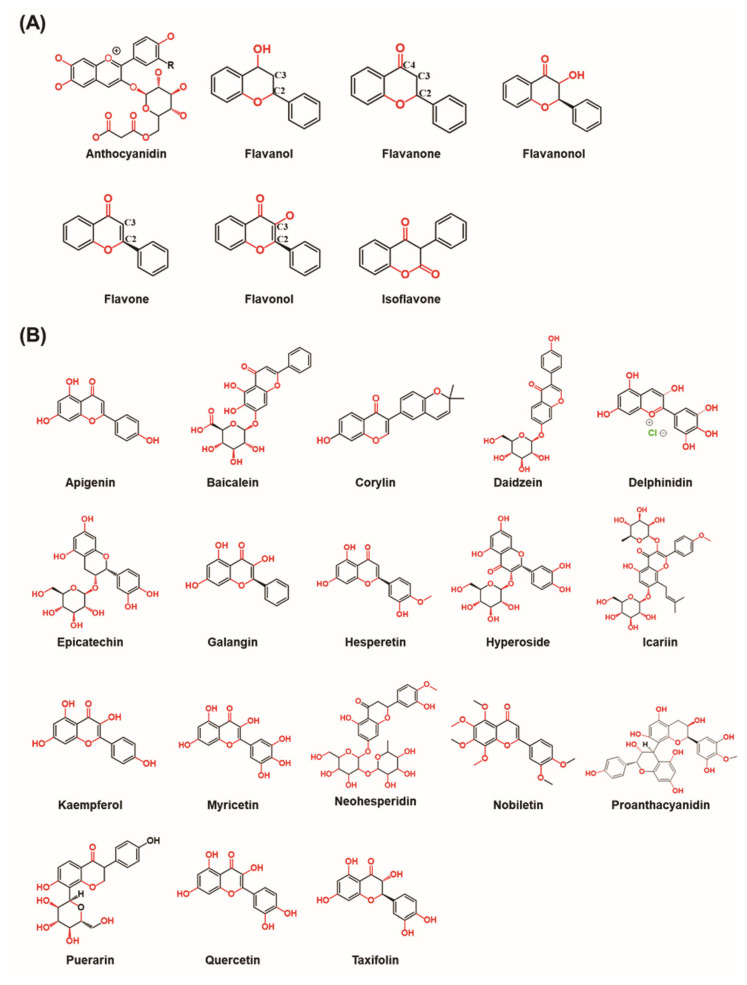
The chemical structures of flavonoids. (**A**) The chemical structure of different types of flavonoids. (**B**) The chemical structure of flavonoids involved in the different bone–related signaling pathways. (Chemical structures source: PubChem (NCBI, NIH, USA; https://pubchem.ncbi.nlm.nih.gov/) accessed on 7 February 2023).

There are seven classes of flavonoids, namely anthocyanidins, flavanols, flavanones, flavanonols, flavones, flavonols, and isoflavones (Figure 1A).

### 2.1. Anthocyanidins

Anthocyanidins are purple, blue, or red pigments in many foods, vegetables, and fruits, particularly in berries such as bilberries, blackberries, raspberries, blueberries, grapes, etc. [30,31]. Red or blue pigments of the anthocyanidins depend on their acid-base balance. Anthocyanin’s appearance varies in different conditions. For instance, it is red in acidic conditions, while blue pigment exists in alkaline conditions. It carries a positive charge in the C-ring oxygen called the flavylium or 2-phenylchromenylium ion. The stability of anthocyanin depends on its structure, pH, light, and temperature [30,32]. Anthocyanin is mainly synthesized with glucose, galactose, and rhamnose in natural products. According to the different substituent groups on the flavylium ring, anthocyanins can be differentiated into each other, such as Delphinidin, Petunidin, Pelargonidin, Cyanidin, Peonidin, and Malvidin [33].

### 2.2. Flavanols

Flavanol monomers known as procyanidins are abundant in the tea plant’s leaves, cocoas, apples, grapes, and wine. An (−)-epicatechin (EC), (−)-epigallocatechin (EGC), (−)-EC-gallate (ECG), and (−)-EGC-3-gallate (EGCG) are involved in the foremost flavanols [34,35]. Catechins are the most readily absorbable flavonoids because they are the only form not bound to sugars (flavonoid glycosides are more easily absorbed after transformation in a glycan form). The flavonol chemical structure has a hydroxyl group on C3 and no double bond between C2 and C3 [36,37].

### 2.3. Flavanones

Flavanones are capable of attaching to various kinds of receptors in the body. Owing to this potential, they are remarked as “privileged structures” and represent various biological reactions [38]. Citrus juices derived from blond or blood and sour oranges, limes, grapefruits, lemons, mandarins, and tangerines belong to the natural sources of flavanones [39]. Hesperidin and Naringin, such as citrus flavanone glycosides, are mostly found in Yuja peel [40]. The chemical structure is remarked by the absence of the ketone group in the C4 position and the presence of a double bond between C2 and C3.

### 2.4. Flavanonols

Flavanonols are the 3-hydroxy derivatives of flavanones and are also referred to as dihydroflavanonols [41]. Flavanonols are not abundant in plants and plant parts that are used for the human diet and are commonly found in wood as free aglycones [42].

### 2.5. Flavones

Flavones broadly exist as glucosides in leaves, flowers, and fruits. The common sources of flavones are celery, parsley, citrus fruits, chamomile, mint, and vegetables. This subclass of flavonoids comprises compounds such as Luteolin, Apigenin, and Tangeritin. Moreover, polymethoxylated flavones such as Tangeretin, Sinensetin, and Nobiletin are found mostly in the peels of citrus fruits [43]. The differentiation between other flavonoids and flavones is a double bond between C2 and C3, which have no alternative to the C3 in flavonoid chemical structure. The oxidation process takes place in the C4 position [44].

### 2.6. Flavonols

Flavonols are also one of the most well-known subclasses of flavonoids. There are two types of flavonols: aglycone form, which is not connected to sugar moieties, and flavonol glycosides, which connect to sugar moieties [45]. Flavonols are pale yellow or colorless compounds that have been shown to impact anthocyanin-mediated coloration by co-pigmentation effects [46,47]. Flavonols are mostly found in fruits and vegetables such as apples, grapes, berries, onions, lettuce, and tomatoes. In addition, flavonols are abundant in tea and red wine. Kaempferol, Quercetin, Myricetin, and Fisetin are the most commonly recognized flavonols [43]. Flavonols chemical structure is remarked by the hydroxyl group in the C3 position [48].

### 2.7. Isoflavones

Isoflavones belong to a subclass of flavonoids that contain phytoestrogen chemicals generated from plants with estrogenic activity [49]. Bioflavonoids, called soybean isoflavones, can interact with a variety of hormones, including estrogen, and they are found in some plants and soy products. It has a molecular structure comparable to estrogen and exists in nature as a molecule with a polyphenolic hydroxyl group called β-glucoside and can be induced by estrogen. Owing to the antiestrogenic and estrogenic activities, it is often referred to as a selective estrogen receptor modulator [50,51]. These compounds may be protective against osteoporosis due to their ability to exert osteogenic and antiresorptive actions on bone, particularly on bone turnover and growth [52,53].

Some of the flavonoids, along with their effect on the bone, are listed in Table 1.

## 3. Bone Signaling Mechanism Affected by Flavonoids

Many studies have demonstrated that flavonoids can induce osteoblast differentiation/proliferation and inhibit osteoclast differentiation/proliferation. The mechanism of action includes the expressions of cytokines, transcription factors, bone-specific matrix proteins, bone signaling pathways, and receptor activators of nuclear factors κB ligand (RANKL)/osteoprotegerin (OPG) system hormone-like biological mechanisms [51]. The most important bone signaling pathways that can be targeted for stimulating bone formation are wingless-related integration site (Wnt)/β-catenin, bone morphogenetic protein (BMP)/transforming growth factor-beta (TGF-β) signaling, mitogen-activated protein kinase (MAPK) signaling, reactive oxygen species (ROS) signaling, nuclear factor kappa-light-chain-enhancer of activated B cells (NFκB) signaling and inflammatory NFκB/nuclear factor of activated T cell c1 (NFATc-1) signaling. The chemical structures of flavonoids involved in different bone–related signaling pathways are illustrated in Figure 1B.

### 3.1. Wnt/β-Catenin Signaling Pathway

Wnt/β-catenin signaling pathway regulates numerous physiological events in many organs, tissues and during growth and development, varying from functions of cell determination, polarity, migration, differentiation, and proliferation [96]. It can be segregated into two groups namely β-catenin (canonical or, β-catenin-dependent) and the non-canonical pathways (β-catenin-independent). The canonical pathway is one of the most important pathways responsible for fracture healing and bone homeostasis. [42].

The Wnt/β-catenin signaling pathway comprises a family of essential proteins for both embryonic development and homeostasis of adult tissues [97]. Wnt proteins, including Wnt3a, Wnt1, and Wnt5a, play a major role in transmitting extracellular signals. The Wnt receptors lipoprotein receptor-related protein (LRP) 5/6 and Frizzled (a unique sevenfold transmembrane receptor Frizzled protein: FZD) are primarily found embedded in the cell membrane. The glycogen synthase kinase-3 (GSK-3) complex, β-catenin, disheveled proteins (DVL), axis inhibition protein (AXIN), adenomatous polyposis coli (APC), and casein kinase 1 (CK1) make up most of the cytoplasmic cascade of Wnt signaling. T-cell factor/lymphoid enhancing factor (TCF/LEF) family members of the β-catenin downstream target gene family include matrix Metalloproteinases (MMPs) and c-Myc, and β-catenin (which translocate to the nucleus) are the key components of the nuclear cascade of Wnt signaling [98]. Once the Wnt ligand binds to its receptor, most DVL protein moves toward the plasma membrane. The clustering of LRP6 and FZD, including the phosphorylation of LRP6, is directed by activated DVL [99,100,101]. Additionally, AXIN and GSK-3β are attracted to the plasma membrane by activated DVL, where they are inhibited from functioning (ubiquitinated degradation of β-catenin) [100,102]. Stabilization of β-catenin in the cytoplasm leads to its nuclear translocation, and it then acts as a coactivator of TCF/LEF transcription factors leading to gene transduction [103,104].

**Figure 2:**
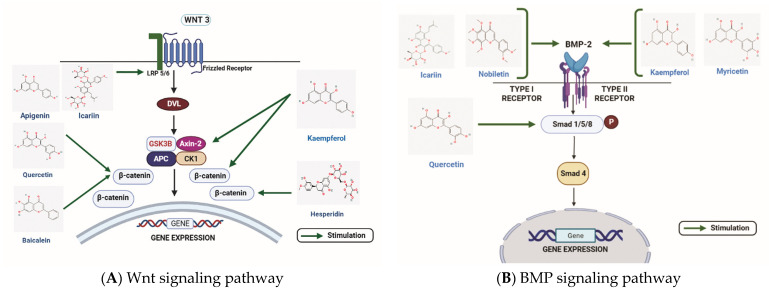
The role of flavonoids in two major signaling pathways inducing bone formation. (**A**) The figure illustrates the stimulatory effects of quercetin, hesperidin, corylin, icariin, apigenin, baicalein on the Wnt/β-catenin signaling pathway. (**B**) Some of the flavonoids such as icariin, nobiletin, myricetin, and kaempferol stimulate osteogenic differentiation by stimulating the BMP2, whereas quercetin targets the Smad 1/5/8 molecule involved in the BMP signaling pathway for stimulating osteogenesis. WNT: Wingless-related integration site; DVL: Disheveled; LRP 5/6: Lipoprotein receptor-related protein 5/6; GSK3β: glycogen synthase kinase 3 beta ; Axin-2: Axis Inhibition protein-2; APC: Adenomatous polyposis coli; CK1: casein kinase 1; BMP: Bone morphogenetic protein; Smad: Suppressor of mothers against decapentaplegic. (Chemical structures source: PubChem (NCBI, NIH, USA; https://pubchem.ncbi.nlm.nih.gov/) accessed on 7 February 2023, Figures created with BioRender.com)).

Various types of flavonoids have the ability to affect the Wnt signaling pathway to alter osteoblast differentiation/proliferation (Figure 2A). Tian X. et al. reported that flavonoid Baicalein could enhance the osteogenic differentiation of tendon-derived stem cells by the induction of the Wnt/β-catenin signaling pathway. The involvement of Wnt/β-catenin signaling was validated by the treatment of DKK-1 (Wnt signaling inhibitor), which reduced the effect of Baicalein on osteogenic differentiation [105]. Moreover, Baicalein was shown to enhance osteogenic differentiation in the pre-osteoblastic cell line, MC3T3-E1, by activating Wnt signaling through MEK/ERK signaling [106]. In another study, Icariin was found to stimulate human bone marrow mesenchymal stem cells (BMSCs) osteogenic differentiation via activation of Wnt signaling. Icariin increased the expression of low-density LRP5, TCF1, and β-catenin. Icariin-activated Wnt signaling and inhibited adipogenesis by regulating the expression of miR-23a [107]. In the rat femoral fracture model, Pan F.F. et al. showed that aApigenin stimulates the osteogenesis of mesenchymal stem cells (MSCs) by increasing the expression of LRP5 and FZD receptors, elevating the level of β-catenin. Apigenin restored the inhibition of osteogenesis when the expression of β-catenin was inhibited by small interfering RNA [108].

Sharma A.R. et al. reported that Kaempferol activated Wnt signaling to induce osteogenesis in the human osteoblast cell line, SaOS-2. Involvement of Wnt signaling was confirmed by inhibiting the expression of β-catenin by its specific inhibitor, FH535. The effect of Kaempferol was further confirmed in primary human osteoblasts and drill-hole mice model. As observed in SaOS-2, both osteogenic models showed induction of β-catenin after Kaempferol treatment [20]. Similarly, Quercetin was shown to promote the protein expression levels of Wnt3 and β-catenin in osteoblasts. Pretreatment of Quercetin rescued the Lipopolysaccharide (LPS)-induced apoptosis and suppressive effect on the osteogenesis of MC3T3-E1 cells. The protective effect of Quercetin was abolished after the pretreatment of MAPK inhibitors or the Wnt/β-catenin inhibitor XAV939 [109]. Likewise, Quercetin was shown to protect TNFα induced inhibition of osteoblast differentiation by inactivation NFĸB and degradation of β-catenin in rat BMSCs [110].

Another flavonoid, Hesperidin, can promote differentiation of alveolar osteoblast cells via activation of Wnt signaling, and it was induced by increasing the expression of β-catenin and cyclin D1. After treating with a Wnt signaling inhibitor, DKK-1, Hesperidin-induced expression of β-catenin and cyclin D1 was decreased, proving Hesperidin’s role in activating Wnt signaling [111]. Chang Y.W. et al. studied the Neohesperidin effect on osteogenic differentiation in BMSCs. Neohesperidin stimulated the Wnt signaling by inducing the expression of β-catenin expression. The use of Wnt signaling inhibitors, DKK1 and XAV939, confirmed the effect of Neohesperidin. In treatment with Wnt signaling inhibitors, Neohesperidin-induced β-catenin expression was decreased [112].

Yu A.X. et al. reported that Corylin could induce osteoblast differentiation on primary osteoblast from calvaria of rats via Wnt signaling. Treatment of Corylin increased the rate of phosphorylation of GSK-3β, promoting Wnt signaling, while the treatment of antagonists such as DKK1 blocked its effect on osteogenesis [75]. In dexamethasone (DEX)-induced osteoporosis mouse model, the role of EGCG on osteogenesis was elucidated. Treatment of EGCG considerably increased the expression of cyclin D1 and β-catenin, stimulating Wnt signaling [113].

### 3.2. BMP/TGF-β Signaling Pathway

BMPs are multifunctional growth factors belonging to the TGF-β superfamily. These proteins represent their fundamental roles in bone repair and skeletal development by interacting with a tetrameric receptor complex leading to intracellular signal transduction with the help of the suppressor of mothers against decapentaplegic (Smad) proteins and expressing the osteoclastogenic genes with the help of transcription factor, runt-related transcription factor 2 (RUNX2) [27]. The noncanonical-Smad-independent pathway is another mechanism involved in TGF-β and BMP2-mediated osteogenesis, resulting in the phosphorylation and activity of RUNX2 [114].

It is well-known that the Smads proteins function as transcription factors and are essential intracellular effectors for BMP and TGF-β family members that influence osteoblast and osteoclast activities [115]. TGFβ or BMP ligands connect to particular type II receptors to attract the associated type I receptor and start a chain of events that phosphorylates their particular Smad receptor (R-Smads). Smad2 and Smad3 are typically required for TGFβ signaling, whereas Smad1, 5, and 8 are required for BMP signaling. The phosphorylated R-Smad and Smad4, the shared partner Smad, come together to form a heterocomplex (Co-Smad). The R-Smad/Co-Smad complex subsequently moves into the nucleus, where it attaches to target genes’ promoters to control the transcription of certain osteoblastic genes [116].

Various studies have highlighted the role of flavonoids in affecting the BMP/TGF- β signaling pathway (Figure 2B). Pang Y. et al. reported that Nobiletin could stimulate osteogenic differentiation by BMP signaling in MG-63 cells. Treatment of Nobiletin induced the expression of BMP2 in a dose and time-dependent manner, elevating the expression of RUNX2 and leading to induction in osteogenic differentiation [78]. Moreover, Icariin could reverse vancomycin-induced inhibition of osteogenesis of rat calvarial osteoblasts. After treatment with vancomycin BMP2 and *RUNX2*, mRNA expressions were reduced, but Icariin co-treatment with vancomycin was able to rescue BMP2 and RUNX expressions [117]. Adhikary S. et al. showed that Kaempferol could reverse the effect of glucocorticoid-induced bone loss on rat calvarial osteoblast cells in vitro and in vivo. The study concluded that glucocorticoid treatment reduced the expressions of RUNX2, BMP2, and BMP4, which was reversed after the treatment of Kaempferol. Similarly, DEX was used to confirm the effect of Kaempferol on BMP signaling. DEX reduced the expressions of RUNX2 and BMP2, but the treatment of kaempferol reversed their expression levels. In addition, Smad1/5/8 phosphorylation was decreased with DEX, followed by the suppressed stimulation of RUNX2 and osteoblast proliferation [118].

Furthermore, Quercetin which chemically resembles estrogen, could induce osteogenesis in BMSCs, as was evidenced by the increased expression of RUNX2, osterix, and osteopontin. The treatment of ICI1827280 (ER inhibitor) to BMSCs was used to validate the presence of estrogen signaling. BMP2, Smad1, Smad4, and p-Smad1 expressions were inhibited by ICI182780, highlighting Quercetin’s role in inducing BMSC differentiation through BMP and ER signaling [119]. Kim H.Y. et al.’s result elucidated that Myricetin could induce osteoblast differentiation in human periodontal ligament cells via BMP signaling along with Wnt/β-catenin and MAPK signaling pathways. BMP2, phosphorylation of Smad1/5/9, and BMP receptor IB levels were increased after treatment with Myricetin, which resulted in the stimulation of osteogenic-related proteins RUNX2 and osterix [120]. Moreover, evidence also suggests that Isoquercetin could induce cell proliferation of BMSCs via BMP signaling. BMP4 was stimulated after treatment with Isoquercetin, and Noggin (BMP antagonist) was able to inhibit the BMP signaling induced by Isoquercetin [121].

In brief, the flavonoids, namely Kaempferol, Nobiletin, Icariin, and Myricetin, target the BMP receptor, whereas Quercetin targets the Smad 1/5/8 molecule involved in the BMP signaling pathway.

### 3.3. MAPK Signaling Pathway

MAPKs are composed of a messenger’s family, which transports various signals from the cell surface to the nucleus, depending on different stimulants such as stress, hormones, and chemicals [122]. Cell migration, differentiation, and proliferation can be regulated by MAPK signaling. The extracellular-signal-regulated kinase (ERK), c-Jun N-amino-terminal kinase (JNK), and P38 are the key members of the MAPK signaling [123].

In multicellular organisms, controlling cell proliferation is a complicated process mostly mediated by external growth factors mediated by the neighboring cells [124]. In order to control cell proliferation, many protein kinases cascades, known as MAPK pathways, are essential [125,126,127]. In order to activate or deactivate their target, mitogen-activated protein kinases phosphorylate either their dual threonine and serine residues (autophosphorylation) or those present on their substrates. As a result, MAPKs control crucial cellular functions such as immune defense, stress reactions, and apoptosis. A MAP3K stimulates a MAP2K, which activates a MAPK in a MAPK module [128,129,130,131,132]. MAPK protein phosphatases (MKPs) dephosphorylate phosphotyrosine and phosphothreonine residues on MAPKs and can inhibit MAPK phosphorylation processes [126,128,133]. The ERK1/2, JNK1/2/3, and the p38 MAPK α, β, δ pathways are three well-known MAPK pathways in mammalian cells. According to their structure, activation motif, and functional forms, they are categorized as ERK, p38, and JNK isoforms [133,134,135]. Growth factors, proinflammatory stimuli, and hormones cause ERK1/2 to become active, while cellular and environmental stressors also cause JNK1/2/3 and p38 MAPK α, β, δ to become active [131,134].

Xing L.Z. et al. examined the effect of Quercitrin on an ovarian-ectomized rat model. After ovariectomization, expressions of osteoblast markers were decreased. Quercitrin increased the expression of the osteogenic marker alkaline phosphatase (ALP) as well as the osteogenic transcriptional factor RUNX2 in the treated ovariectomized rats. Administration of Quercitrin increased the phosphorylated forms of ERK, P38, and JNK in ovariectomized rats, implying that Quercitrin reversed the osteoporosis effect in ovariectomized mice model by employing MAPK signaling [136]. Similarly, Baicalein attenuated osteomyelitis by inhibiting Toll-like receptor 2 (TLR2) and MAPK signaling in *Staphylococcus aureus*-treated mice and MC3T3-E1 cells. Treatment with *Staphylococcus aureus* increased the expressions of TLR2 and MAPK signaling (p-ERK and p-JNK) in treated MC3T3-E1 cells. The knockdown of TLR2 with shRNA reversed the effect of *Staphylococcus aureus* on MC3T3-E1 cells. Baicalin utilized a similar mechanism of inhibition of TLR2 to induce osteogenesis in MC3T3-E1 cells [137].

Xu Q. et al. evaluated the effect of Icariin on RAW 264.7 cells treated with RANKL to induce osteoclastogenesis. Treatment of RAW 264.7 cells with RANKL increased NFĸB and MAPK signaling pathway. The phosphorylated forms of P38, ERK, and JNK were found to be elevated. Treating RAW 264.7 cells with Icariin reversed the RANKL effect of inducing osteoclastogenesis by inhibiting NFĸB and MAPK signaling [138].

Liu H. et al. studied the role of Hesperetin on LPS-induced osteoporosis. Hesperetin rescued the osteoclastogenesis-inducing effect of RANKL in RAW 264.7 cells. Hesperetin mediated effect was due to the inhibition of NFĸB and MAPK signaling pathways in RAW 264.7 cells. Similar to in vitro results, Hesperetin rescued LPS-induced bone loss, decreased osteoclast numbers, and suppressed the RANKL/OPG ratio in mice [139]. In addition, Kaempferol can also stimulate osteoblastogenesis in MC3T3-E1 cells treated with DEX by activating MAPK signaling. Treatment with DEX suppressed the expression of RUNX2, Osterix, and ALP activity. However, treatment of Kaempferol attenuates the DEX-induced inhibition of osteogenesis. In the MC3T3-E1 cells treated with DEX, p-P38 decreased significantly, but no significant changes were observed in p-JNK. However, after treatment with Kaempferol, both the phosphorylated forms were shown to increase [140].

### 3.4. Antioxidant/ROS

The ROS molecule contains unstable oxygen, which can affect other molecules in the cell. When ROS is less, these molecules are able to mediate several signals for cell differentiation, proliferation, and self-renewability. On the contrary, excess ROS will increase oxidative stress following an imbalance in normal tissue homeostasis, resulting in poor tissue management and wound healing. Oxidative stress can lead to cell death and damage to the proteins and nucleic acid [141,142]. In mitochondria, ROS are mainly produced in the electron transport chain as oxidative phosphorylation byproducts [143].

Enzymes produce intracellular ROS, generally as O_2_, hydrogen peroxide (H_2_O_2_), and OH [144,145,146]. H_2_O_2_ functions as a second messenger that can integrate environmental cues, swiftly diffuse through membranes, trigger downstream signal transduction cascades, and have a variety of downstream destinations [146,147]. Studies have also elucidated that the family of MAPK, such as p38 MAPK and ERK1/2, are some of the well-known downstream effector molecules of the ROS and usually play an important role in the differentiation of osteoblasts [148,149], probably by activating the p38 MAPK and ERK1/2 pathways. However, more detailed studies are required to ascertain these facts [150].

**Figure 3:**
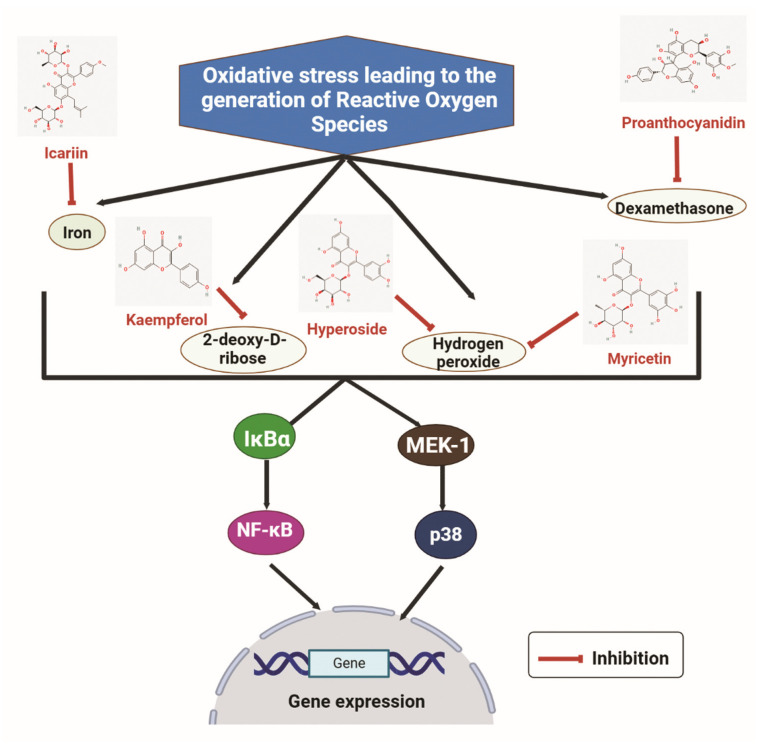
The inhibitory effects of some flavonoids such as icariin, kaempferol, hyperoside, myricitrin and proanthocyanidin on some reactive oxygen species (iron, dexamethasone, hydrogen peroxide, and 2-deoxy-D-ribose). IκBα: Inhibitor of nuclear factor kappa B alpha; NF-κB: Nuclear factor kappa B; MEK-1: Mitogen-activated protein kinase-1. (Chemical structures source: PubChem (NCBI, NIH, USA; https://pubchem.ncbi.nlm.nih.gov/) accessed on 7 February 2023, Figures created with BioRender.com).

Flavonoids have high antioxidant activity against ROS and have been reported to rescue the negative effect of ROS on bone. The effect of different flavonoids on the ROS, namely iron, DEX, H_2_O_2_, and 2-deoxy-D-ribose (dRib), are shown in Figure 3. Icariin can suppress oxidative stress induced by iron on MC3T3-E1 cells. The viability of MC3T3-E1 cells was decreased after treatment with iron chloride. ROS production was induced by iron chloride. Nevertheless, cell viability was increased after treatment with Icariin, and the ROS production was inhibited dose-dependently. Furthermore, osteogenic differentiation markers such as RUNX2 and Osterix were significantly increased after Icariin treatment. Moreover, iron chloride stimulated osteoclast formation, but the treatment of Icariin inhibited it [151]. Similarly, the effect of Kaempferol was studied on MC3T3-E1 cells treated with dRib. dRib produces oxidative stress through autooxidation and glycosylation. The treatment of dRib notably decreased cell viability, collagen content, and mineralization of MC3T3-E1 cells, but Kaempferol significantly reversed the dRib-induced effects in osteoblasts. Moreover, the treatment of dRib to MC3T3-E1 cells increased the levels of Malondialdehyde (MDA), an indicator for ROS. However, the increased MDA level by dRib was reversed by Kaempferol [152].

Qi X.C. et al. reported that another flavonoid Hyperoside (Quercetin 3-O-β-D-galactose) could protect against the oxidative stress induced by H_2_O_2_ in MC3T3-E1 cells. Hyperoside decreased apoptosis induced by H_2_O_2_ and rescued the osteogenesis-related markers collagen I and osteocalcin (OCN). Additionally, H_2_O_2_ treatment increased MAPK signaling (p-P38 and p-JNK) to inhibit the effect of H_2_O_2_, concluding that oxidative stress induced by H_2_O_2_ can be reversed by the antioxidant property of Hyperoside [153]. Huang Q. et al. showed the effect of Myricitrin on oxidative stress induced by H_2_O_2_ on human BMSCs and ovariectomy-induced osteoporosis model. Treatment of H_2_O_2_ results in decreased cell viability, mineralization, and expression of osteogenic markers ALP and OCN. However, the application of Myricitrin reversed the effect of H_2_O_2_ in human BMSCs. In the ovariectomized mice model, increased levels of MDA (end product of lipid peroxidation) and reduced glutathione (intracellular antioxidant) are observed. Treating Myricitrin to ovariectomized mice model attenuated the oxidative effect of H_2_O_2_ [154].

Chen L. et al. demonstrated that Proanthocyanidins (PAC) could suppress oxidative stress in primary osteoblasts induced by DEX. DEX-treatment induces cell apoptosis in osteoblasts by enhancing the expression of several apoptotic markers such as Caspase 3 and Bax. However, PAC reversed the DEX effect, stimulated Bcl2 expression, and inhibited Bax and Caspase 3. Moreover, DEX inhibited the Nrf2 transcription factor related to the regulation of oxidative stress. The effect of PAC was analyzed in the DEX-treated rat model. Nrf2 expression was higher in DEX-PAC treated model compared to the only DEX-treated one [155].

### 3.5. NFκB/NFATc-1 Signaling

NFκB is one of the most important regulators involved in bone remodeling and inflammation. The NFκB activity is promoted when the rate of bone resorption exceeds bone formation [156]. RelA (p65), p52, c-Rel, RelB, and p50 are the key members of NFκB signaling. The canonical p65/p50 and NFκB heterodimeric complex is a prevalent isoform of NFκB. Several pro-inflammatory stimulants such as Interleukin (IL)-1 and TNF can stimulate the NFκB pathway. However, the non-canonical NFκB pathway is induced by releasing a small subgroup of TNF family members [157].

The canonical NFκB pathway reacts to a variety of stimuli, such as ligands for pattern-recognition receptors (PRRs), various cytokine receptors, members of the TNF receptor (TNFR) superfamily, and T-cell and B-cell receptors [158]. The inducible degradation of inhibitor of nuclear factor kappa B alpha (IκBα), which is brought on by its site-specific phosphorylation by a multi-subunit IκB kinase (IKK) complex, is the main mechanism for canonical NFκB activation [159,160]. Two catalytic subunits, namely IKKα and IKKβ, as well as a regulatory component known as NFκB essential modulator (NEMO) or IKKγ, constitute the IKK molecule [161]. Various stimuli, such as cytokines, growth factors, mitogens, microbial components, and stressors, can activate IKK [162]. When IKK is activated, it phosphorylates IκBα at two N-terminal serines, which causes IκBα to be degraded in the proteasome in a ubiquitin-dependent manner. This causes the nuclear translocation of canonical NFκB members, primarily the p50/RelA and p50/c-Rel dimers, to occur very quickly and causes gene transduction [160,163,164].

**Figure 4:**
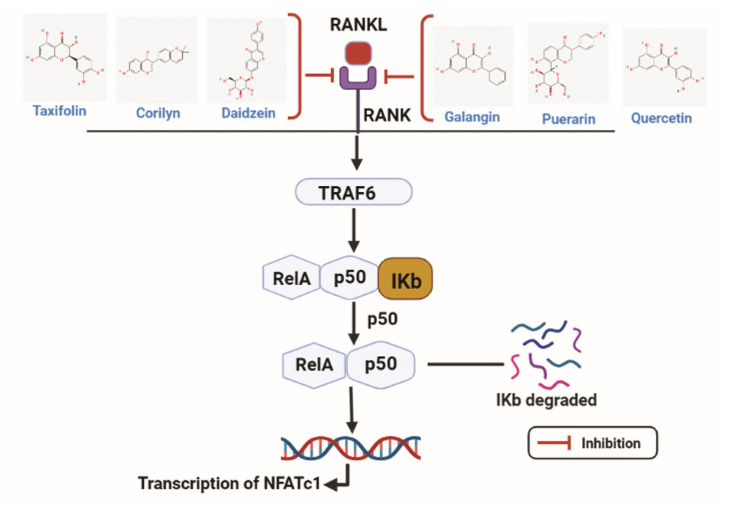
Taxifolin, corilyn, daidzin, galangix, puerarin, and quercetin acts as antagonists by hindering the transcription of the NFATc-1 gene by targeting RANKL. RANKL: Receptor activator of nuclear factor kappa-Β ligand; RANK: Receptor activator of nuclear factor kappa-Β; TRAF6: Tumor necrosis factor receptor associated factor 6; NFATc1: Nuclear factor of activated T cells 1. (Chemical structures source: PubChem (NCBI, NIH, USA; https://pubchem.ncbi.nlm.nih.gov/) accessed on 7 February 2023, Figures created with BioRender.com).

Flavonoids have a role in effectively hindering the transcription of the *NFATc-1* gene by targeting RANKL (Figure 4). Xiao L. et al. studied the effect of Puerarin on the differentiation of osteoclasts where RANKL was treated to RAW 264.7 cells. TRAF6 and ROS expressions were elevated in RANKL-stimulated RAW 264.7 cells, and the Puerarin treatment reversed the effect. Furthermore, NFκB and MAPK signaling were related to the effect of Peurarin on RANKL treatment. The phosphorylated form of p65 and IĸBα expressions were increased with RANKL treatment, followed by the increase in p-p38, p-JNK, and p-ERK. After treatment with puerarin, phosphorylated forms of NFĸb and MAPK signaling were significantly decreased. Puerarin inhibited osteoclastogenesis by suppressing NFĸb and MAPK signaling [165]. Alternatively, the flavonoid molecule Epicatechin 3-O-β-D-allopyranoside (ECAP) has an anti-osteoclastogenesis effect on RANKL-stimulated RAW 264.7 cells. RANKL treatment activates the NFATc-1 and NFκB, which is important for osteoclastogenesis. ECAP altered the effect of osteoclastogenesis induced by RANKL by downregulating the phosphorylation of p65, IĸBα, as well as NFATc-1 expression dose-dependently [65].

Similarly, Hyperoside can protect against ovariectomy-induced bone loss. The bone resorption markers, namely C-terminal telopeptide of type I collagen (CTX) and tartrate-resistant acid phosphatase 5b (TRAP-5b) were increased after ovarian resection, but treatment with Hyperoside resulted in decreased bone resorption. In ovariectomized mice model p-p65, p-IĸBα and NFATc-1 expression levels were increased, which was further attenuated by Hyperoside. These results indicate that NFκB and NFATc-1 signaling pathways are related to the mechanism of Hyperoside in the ovariectomy-induced osteoporosis mice model [166]. EGCG can suppress osteoclastogenesis by inhibiting osteoclast-specific markers as well as NFκB and MAPK signaling in RANKL-stimulated RAW 264.7 cells [167].

Studies elucidated the effect of Daidzin on RANKL stimulation on the BMSCs. Daidzin can protect osteoclastogenesis on bone marrow-derived macrophages (BMMs) by inhibiting osteoclast-specific transcript factors such as NFATc-1, c-Fos, TRAP, CTSK, and the NFκB signaling pathway [168]. Lastly, Delphinidin-enriched maqui berry extract (MBE) was shown to improve the osteogenic activity of MC3T3-E1 cells. MBE inhibited the transnucleation of p65 induced by LPS. The osteoclastogenesis effect was evaluated by RANKL treatment on primary mouse bone marrow cells. The NFATc-1 and CTSK were increased with RANKL but time-dependently reversed with MBE treatment. Therefore, MBE can be associated with osteoblastogenesis by impeding the NFκB signaling pathway [169]. Zhang H.Q. et al. indicated that Taxifolin could suppress osteoclast differentiation induced by RANKL on BMMs. mRNA expressions of osteoclast markers such as *TRAP*, *NFATc-1*, and *CTSK* were increased with RANKL treatment, whereas treatment of Taxifolin reversed the expressions in a dose-dependent manner [170].

### 3.6. Other Signaling Pathways

Numerous studies reported the effects of flavonoids on osteoblastogenesis and osteoclastogenesis through many signaling pathways.

Zhou L. et al. examined Icariin’s effect in the ovariectomized rat model, MC3T3-E1 cells, and BMSCs. BMSCs isolated from the ovariectomized rat model shows a decrease in ALP and enhanced osteoclastogenesis. Icariin treatment reversed the effect similarly with a positive control E2 (phytoestrogen). Insulin-like growth factor I (IGF-I) signaling can activate estrogen receptors by stimulating phosphoinositide 3-kinase (PI3K). Icariin treatment stimulated the levels of ERα and IGF-IR on BMSCs. In MC3T3-E1 cells, Icariin stimulated the expression of IGF-IR, but after blocking the IGF-IR with PPP (IGF-IR kinase inhibitor picropodophyllin), the Icariin effect was inhibited. Moreover, Icariin can stimulate bone formation by IGF-I1 and ER signaling in the ovariectomy-induced osteoporosis model [171]. Additionally, Icariin treatment elevated collagen 1-α1, OCN, and ALP levels. Notch signaling molecules were suppressed by Icariin treatment, such as Hes1 and Hey1. Moreover, inhibiting Notch signaling with DAPT (N-[N-(3, 5-difluorophenacetyl)-l-alanyl]-s-phenylglycinet-butyl ester), a γ-secretase inhibitor, enhanced the effect of Icariin. Ovariectomized mice model had increased expression levels of NFATc-1 and Notch 1, which was attenuated by Icariin, indicating its role in osteoblastogenesis by regulating Notch signaling [172]. Icariin also plays a major role in osteoblastogenesis by activating cAMP signaling in rat calvarial osteoblasts. Icariin treatment increased ALP activity and cAMP contents with an elevation in the level of p-PKA and p-CREB [173].

Another flavonoid, Luteoloside, is shown to inhibit osteoclastogenesis on RANKL-stimulated BMMs. Luteoloside inhibited the osteoclast markers NFATc-1, CTSK, and calcitonin dose-dependently. RANKL induced the Ca^2+^ oscillation and NFATc-1, but Luteoloside rescued the effect. Treatment of Luteoloside also reversed the effect of MAPK signaling. Activation of NFkB was significantly reduced with the Luteoloside treatment. Luteoloside also suppressed RANKL-induced osteoclastogenesis through NFATc-1 and Ca^2+^ signaling, as well as NFĸB and MAPK signaling [174]. Cai P. et al. reported the effect of Baicalein on MC3T3-E1 cells treated with glucocorticoid. Baicalein directly targeted genes were Ak strain transforming (AKT) 1, forkhead box protein O (FOXO) 1, and FOXO3. The glucocorticoid treatment decreased the levels of several osteoblastogenesis markers, namely ALP, RUNX2, and OCN. Treatment of Baicalein attenuated glucocorticoid’s effect, inhibited the p-AKT expression level, and stimulated the FOXO1 expression. Silencing of the *AKT* with *siRNA-AKT* increased FOXO1, suppressing OCN, ALP, and RUNX2. All evidence shows that Baicalein can inhibit glucocorticoid-induced osteoporosis by regulating AKT/FOXO1 signaling [175].

Similarly, Kaempferol can induce osteoblastogenesis by mTOR signaling on BMSCs from the ovariectomized mice model. BMSCs from ovariectomized mice have decreased osteoblastogenesis markers ALP, RUNX2, and osterix, but the treatment of Kaempferol increased their expressions. Rapamycin, an mTOR inhibitor, was used to assess the effect of Kaempferol. The downstream regulators of mTOR, 4E/BP1, and S6K1 concentrations were induced in the ovariectomized mice model, but Kaempferol reversed the effect indicating its potency to stimulate osteoblast differentiation on ovariectomized BMSCs by regulating mTOR signaling [176]. Zhou Y. et al. demonstrated that Puerarin stimulated osteoblastogenesis in BMSCs via p53/TNFα/STAT1 signaling. Treatment with Puerarin suppressed the pro-inflammatory cytokines such as IL-6, TNFα followed by the increased production of anti-inflammatory cytokines such as IL-2 and IL-10. Apoptotic markers such as caspase 3 and caspase 9 were decreased, followed by the upregulation in miR-155-3p level. Additionally, the levels of TNFα, STAT1, and p53 were decreased. Inhibiting miR-155 increased the TNFα, STAT1, and p53, showing that Puerarin can regulate osteoblastogenesis by inhibiting p53/TNFα/STAT1 signaling [177].

## 4. Absorption of Flavonoids

One of the most pivotal factors responsible for maintaining the normal physiological functions of flavonoid molecules in the body is its bioavailability [178]. In plant diets, flavonoids are primarily found as glycosides. Since they are bonded to sugars in the form of beta-glycosides, flavonoids found in food were formerly thought to be indigestible [43]. The aglycone part penetrates the epithelial cells of the intestine after undergoing the process of hydrolysis, where it is processed by the phase II enzymes to produce the corresponding conjugated metabolites [24]. Following the consumption of flavonoids, the conjugated metabolites are identified in the plasma, as the majority of flavonoids undergo methylation, glucuronidation, and sulfation in the liver and small intestine [178]. Most dietary nutrients are primarily absorbed in the small intestine [27]. The lacteals or portal veins receive the absorbed flavonoids after conjugation through the small intestine. Only the aglycone parts are generally absorbed since the glycosides were deemed extremely hydrophilic for passive diffusion via the small intestine. Thus, only a small amount of glycosylated flavonoids are absorbed. One of the theories for the tea flavonoids’ poor bioavailability and absorption is that they are unstable in the colon [24].

## 5. Delivery System of Flavonoids for Bone

**Figure 5:**
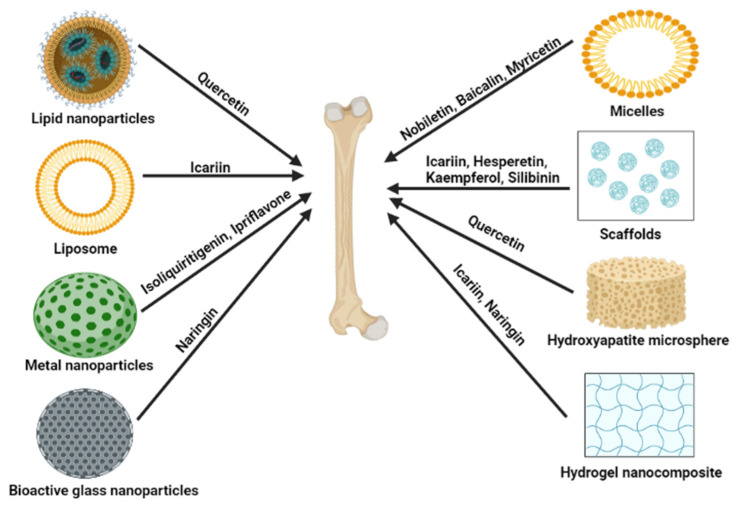
Potential delivery systems for delivering flavonoids to the bone. Figures created with BioRender.com.

The highly unsaturated structure of flavonoids causes reduced potential bioactive benefits by easy oxidation and degrading of flavonoids [179]. Flavonoids as therapeutic agents are more limited applications because of their low bioavailability and aqueous solubility. A strategy to overcome the limitations mentioned above is to apply the delivery system for flavonoids by carriers using chemical and biological methods [180,181]. For example, liposomes formed by lipid and water bilayers can catch both substances that have hydrophilic or hydrophobic properties, respectively. Some structures of flavonoids can indicate significant loading abilities in membranes of liposomes and can affect the properties of encapsulating and stabilizing liposomes by binding structures of loaded flavonoids and liposomes [179,180]. Recent advancements have elucidated the use of lipid nanoparticles, liposomes, micelles, scaffolds, and hydrogen nanocomposite to deliver different flavonoids (Figure 5).

### 5.1. Lipid Nanoparticles

Quercetin-loaded solid lipid nanoparticles (oral administration, 5 mg/kg/day) have higher abilities to alleviate bone loss and enhance bone strength compared to only quercetin treatment in the ovariectomized rat model [182].

### 5.2. Liposome

For delivering the osteogenic phytomolecule to cells, Asp8-liposome can be an efficient delivery system. In an estrogen depletion-induced osteoporosis mice model by ovariectomy, Asp8-icaritin-liposome can prevent osteoporosis by inducing bone formation [183]. Asp8-liposome-Icaritin bone-targeting delivery system effectively prevents steroid-associated osteonecrosis in the rat model. In addition, the Asp8-liposome delivery system also can enhance osteogenesis and inhibits osteoclast activity [184]. Sun X. et al. have developed a novel formulation that is biomineral-binding liposomes (BBL) loaded with icariin as a new therapeutic candidate for osteoporosis. In BBL formulation, pyrophosphate acts as the bone-binding moiety, and drug-conjugated liposome rapidly strongly binds to hydroxyapatite (HA). BBL loaded with Icariin has good therapeutic efficacy on the ovariectomized + glucocorticoid group in the rat model [185].

### 5.3. Metal Nanoparticles

Isoliquiritigenin-encapsulated mesoporous silica nanoparticles (MSNs-ISL) suppress the remarkable RANKL-induced osteoclastogenesis and inhibit osteolysis by osteoclast in BMMs. Therefore, MSNs-ISL can protect against the destruction of bone inflammation [186]. SiO_2_-CaO systems with a hollow core surrounded by mesopores in a radial arrangement are called NanoMBGs. It induces the response of macrophages against stimulation of LPS and IL-4. Moreover, it does not induce the polarization of macrophages regarding the M1 pro-inflammatory phenotype. NanoMBGs loaded with Ipriflavone do not affect cell proliferation and cell viability of osteoblasts in coculture with osteoclasts while remarkably inhibiting the proliferation of osteoclasts and activating resorption [187].

### 5.4. Bioactive Glass Nanoparticles

Mesoporous bioactive glass nanoparticles suppress the inflammatory responses of macrophages. Bioactive glass nanoparticles was modified with β-cyclodextrin and was loaded with Naringin (Aladdin, Shanghai, China) (are called as NG@CD- bioactive glass nanoparticles). NG@CD-bioactive glass nanoparticles facilitates the transformation to the M2 phenotype in macrophages. In addition, NG@CD- bioactive glass nanoparticles synergistically promotes osteogenesis and suppresses osteoclast formation in the local immune microenvironment. In a rat model with a femoral defection, NG@CD-bioactive glass nanoparticles increases the expression level of osteogenesis-related genes and the formation of new bone [188].

### 5.5. Micelles

Nobiletin-PEG-PCL micelles developed by loading Nobiletin to poly(ethylene glycol)-block-poly(e-caprolactone) could increase its circulation time by preventing the rapid release of Nobiletin from micelles. Nobiletin-PEG-PCL restrains osteoclast differentiation in BMMs via the MAPK signal pathway by RANKL. In addition, Nobiletin-PEG-PCL reduces the loss of bone and enhances bone density in ovariectomized mice model [189]. Baicalein encapsulated D-α-tocopherol polyethylene glycol 1000 succinate (TPGS) polymeric micelles (PMs) effectively reduced the damaged gingival fiber and destructed alveolar bone by direct injection in a rat model with periodontal disease [190]. The AL-P(LLA-CL)-PEG-P(LLA-CL)-Myricetin-loaded micelles are expected as a bone-targeting delivery system for the treatment of osteoporosis. In an ovariectomized rat model, AL-P(LLA-CL)-PEG-P(LLA-CL)-Myricetin-loaded micelles showed the improved oral bioavailability of Myricetin and excellent capability for bone-targeting [191]. Self-assembled nanomicelles could act as useful oral carriers to deliver therapeutics with low bioavailability for osteoporosis treatment. Circinal–Icaritin by self-assembled nanomicelles (CIT-SO-DOC) improved the bioavailability of CIT and increased the effect of preventing osteoporosis by reducing the size and enhancing the absorption of CIT [192].

### 5.6. Scaffolds

Porous composite scaffolds with Icariin-loaded HA/alginate (Icariin/HAA) stimulated cell proliferation in BMSCs, meanwhile no cytotoxicity. Especially porous composite scaffolds with icariin/HAA suppressed osteoclast activation in vivo. Moreover, it enhanced gene expression levels of osteogenic markers and the Wnt signaling-related genes. Further, bone regeneration of rabbits with radius bone defection was improved by porous composite scaffolds with Icariin/HAA [193].

Composite hierarchical porous scaffolds were developed for carrying the BMP2 and Icariin under a controlled drug delivery system (SFBMP2/SBA15IC) and composed of silk fibroin micropores and SBA15 mesopores (SF/SBA15). SFBMP2/SBA15IC has an excellent induction ability of osteogenesis than other BMP2 or Icariin-loaded groups by showing higher increased osteogenic differentiation gene expression and ALP staining and mineralization in co-cultured BMSCs [194].

Calcium phosphate cement (CPC) scaffolds loaded with Icariin were synthesized for successfully delivering Icariin. Icariin enhances the osteogenic and angiogenic effects in BMSCs and inhibits osteoclastogenesis. CPC scaffolds are a better delivery system for repairing bone defects because of their capability to improve osteogenesis and angiogenesis. Thereby, systemic administration of CPC scaffolds loaded with Icariin could promote the repair of bone defects and the prevention of osteoporosis in the ovariectomized rat model [194]. Dual drug release is achieved by incorporating Icariin and vancomycin into an injectable CPC. It is a notable potential therapeutic candidate for bone infection disease or contaminated bone injury because Icariin-VA-CPC scaffolds have antibiotic and osteoinductive effects. [195]. Icariin–small-intestine submucosa scaffolds (Icariin-SIS) elevated osteogenic markers such as ALP, bone sialoprotein (BSP), and OCN in MC3T3-E1 cells. The formation of new bone was accelerated after implanting IC-SIS in mice with calvarial defects [196]. Another study reported that icariin–extracellular matrix (ECM)-SIS induced higher expression levels of markers of osteogenic differentiation (such as ALP, BSP, and OCN) and BMP4 expression than ECM-SIS (same as Icariin-SIS). Moreover, Icariin-ECM-SIS implanted mice with defective calvaria have increased bone regeneration and a higher new bone formation ratio compared to ECM-SIS or Icariin-SIS implantation ground [197]. Hesperedin/gel sponge scaffolds successfully carried Hesperedin to human MSCs and induced osteogenic differentiation by activating the ERK and Smad signaling pathways. Hesperedin/gel sponge scaffolds significantly promoted fracture healing of rat tibia in the rat osteotomy model [198]. Layer-by-layer (LBL) nano-matrix, incorporated with Kaempferol characterized as stable, increased drug delivery ability and pharmacokinetics, and inhibited enzyme degradation. Therefore, the LBL nano-matrix incorporated with Kaempferol stimulated osteogenesis in the osteopenic rats [199]. Alginate/gelatin-Silibinin scaffolds could be usefully applicated to bone tissue engineering because they enable continuous release of Silibinin during an extended time and induction of bone formation in vitro [200].

### 5.7. HA Bioceramic Microspheres

For application in carrying Quercetin, nHA bioceramic microspheres developed having a micro–nano hybrid surface. nHA bioceramic microspheres sustained the release of Quercetin. Moreover, nHA bioceramic microspheres with Quercetin could induce the formation of bone and angiogenesis in femur defect rats with ovariectomy [201].

### 5.8. Hydrogel Nanocomposite

Improved alginate dialdehyde–gelatin hydrogel nanocomposite by incorporating mesoporous silica–calcium nanoparticles and Icariin loading assures enhanced osteoblast proliferation, adhesion, and differentiation in MC3T3-E1 cells [202]. The hydrogel loaded with Naringin indicated the rapid release of Naringin at pH 5.5 to 6.5. Cell viability increased at 0.85% after Naringin treatment. Especially, CHC-β-GP-glycerol hydrogel that carries Naringin inhibited loss of periodontal bone and infiltration by inflammation. Further, it meaningfully suppressed the expression of TLR2, RAGE, and TNFα in periodontitis [203].

### 5.9. Phase-Transited Lysozyme (PTL)-Primed Ti Surface

PTL-primed titanium surface can locally deliver Icariin through a layer-by-layer self-assembly system. PTL-primed titanium surface with Icariin-immobilized HA/chitosan multilayer enhances the osteogenesis on osteoblasts and improves early osseointegration in vivo by continuously releasing icariin [204].

### 5.10. Nano Coating

Flavonoids can be coated on the surface of osseo-integrable implants and can be made to release at the desired site of action. The Quercetin was grafted on titanium coins (between 64 ± 10 and 842 ± 361 nmol on 6.2 mm). Quercetin-nanocoated titanium surface promotes mineralization in human BMSCs and can accelerate osteointegration in bone implants [205].

## 6. Conclusions

Diseases associated with the skeletal system can limit an individual’s mobility and can often result in considerable morbidity. The increase in the activity of osteoclasts or decreased activity of osteoblasts is the underlying molecular cause of osteoporosis. This imbalance in the bone remodeling process causes accelerated bone resorption and suppressed bone formation [206]. Moreover, the pathophysiology of osteoporosis is now understood to include decreased bone density and skeletal fragility brought on by several factors, including (1) defects in the trabecular microarchitecture; (2) flaws in the intrinsic materials of the bone tissue; (3) imperfections in the repair of microdamage from routine daily activities; and (4) excessive rates of bone remodeling [207]. Numerous drugs and therapeutical approaches have been tested to establish an efficient cure for osteoporosis [208]. Patients with osteoporosis can employ pharmaceutical treatments to lower their fracture risk and boost bone mineral density. Still, their usage is constrained by the side effects, which depend on a variety of factors, including past medical history, genetics, and patient’s nutritional habits [209,210,211,212,213]. Commercially available drugs for osteoporosis, their signaling mechanism of action, and known side effects are listed in Table 2. The long-term use of commercially available bisphosphonates such as Alendronate, Zoledronate, and Risedronate has been reported to cause some substantial side-effects such as diarrhea, gastritis, nausea, and flatulence [214]. In addition, the implementation of RANKL inhibitors such as Denosumab has potential side effects such as urinary tract infection, polyoma (BK) viremia, and flu-like syndrome compared to the control subjects [215]. The prevention of osteoporosis by hormone-replacement therapy, including estrogen, has the chance of developing cardiovascular diseases, breast cancer, thromboembolic disorders, and stroke [216]. An anti-sclerostin antibody, namely Romosozumab, increases the Wnt signaling activity to maintain a homeostatic balance between bone resorption and bone formation. However, it has been shown to increase the chances of stroke and cardiovascular difficulties in seven patients in the first year of the trial [217]. New advancements in the field of therapeutics against bone diseases are urgently required to effectively treat osteoporosis while reducing side effects and regardless of changing patient-related circumstances [208].

Flavonoids have favorable and preventive effects on the pathological aspect of bone loss and the emergence of osteoporosis. Administering substances that have an impact on bone deposition and remodeling at the same time is one of the potential therapy options using purified chemicals. Dietary supplements consist of a range of natural bioflavonoids as essential components. Flavonoids have been shown to possess various therapeutic potentials such as antitumor, antimicrobial, anti-oxidant, anti-cancer, anti-inflammatory properties, stimulating osteogenic abilities, etc. Some flavonoids, including anthocyanins, flavonols, and isoflavones, have dual osteoclast and osteoblast stimulatory effects [11]. Compared to pharmaceutical treatments, these naturally occurring phytochemicals with powerful bone-conserving qualities beyond vitamin D and calcium have fewer or no adverse effects. In addition to their inherent chemical anti-oxidant abilities, flavonoids are also being researched for their potential anti-inflammatory properties. As discussed in previous sections, some flavonoids such as Cyanidin, Daidzein, Cladrin, Calycosin, Icariin, and Petunidin have been shown to function by activating osteoblasts and inhibiting osteoclasts [11]. Few clinical trials have been carried out, although several flavonoids have demonstrated to have largely preventive properties aimed at preventing pathological bone loss, highlighting their specific effects on osteoblast and osteoclast differentiation and activity through the same interactors. The preventive properties of flavonoids have been shown in a number of preclinical studies. Even though these chemicals definitely play epigenetic roles, only a few chemical studies have elaborated on their mechanism [244]. These bioactive substances are thought to stimulate bone formation and prevent bone resorption by controlling cell signaling pathways such as Wnt and BMP signaling that affects osteoblast and osteoclast development in tandem. For instance, with minimal or no carcinogenic side effects, the bioavailability of isoflavones possessing the selective estrogen receptor affinity can potentially prevent osteoporosis [245].

Additionally, data from a randomized, double-blind, placebo-controlled trial portrayed the beneficial effect of the flavonoid icariin in treating osteoporosis which has comparatively lower chances of causing cardiovascular ailments and breast cancer [246]. Nevertheless, the activities of flavonoids are not explained by a single mechanism but rather by a confluence of numerous routes. Studies currently underway point to the potential for including flavonoids in grafts and bone scaffolds to ensure local administration and continuous release of flavonoids that can speed up bone healing [247].

Numerous studies have studied the interaction of flavonoids with various target proteins involved in osteogenesis. Jiang J. et al. performed molecular docking studies to predict and verify the roles of some flavonoids, Icariin, Baohuside-I, and Icartin, in reversing the inhibitory effect of the glucocorticoid-induced bone formation. These flavonoids were docked with some of the proteins such as RANKL, RUNX2, OPG, BMP2, and BMP4 [248]. Hu Y. et al.’s molecular docking studies also delineated the mechanism of using quercetin against osteoporosis. NFKB1, IL-1β, and RelA had an increased binding potency which mainly explains Quercetin’s anti-osteoporosis activity [249]. Additionally, the role of Kaempferol in curing senile osteoporosis was also confirmed by performing molecular docking studies. The study revealed that Kaempferol is a potent curative agent against this musculoskeletal disease as it can regulate oxidative stress, various inflammatory pathways, and bone homeostasis [250]. For the first time, molecular docking investigations performed by Yu X. et al. revealed that Naringin might treat osteoporotic fracture, presumably via modulating a variety of signaling pathways and targets associated with the development of osteoclasts and oxidative stress. The docking study was performed using 8 proteins, namely SERPINE1, CASP3, PPARG, ESR1, MMP1, TNF, CYP19A1 and ACE [251].

However, poor bioavailability and long-term stability issues have hindered their clinical impact. Recently, with the advent of new-age delivery methods, including polymer science and nanotechnology, the issues with flavonoid bioavailability and toxicity are being resolved. Thus, flavonoids with bone-sparing properties are being considered for bone tissue engineering and regenerative medicine for unmet needs for bone augmentation. Even the inclusion of certain biomaterials to deliver flavonoids can contribute to bone tissue engineering. A synergistic interaction between nanoscience and flavonoids might enable the formation of hybrid nanocomposites to enhance their epidemiological properties. However, researchers should consider each delivery system’s pros and cons before deciding the delivery method for flavonoids for therapeutic purpose (Table 3).

**Figure 6:**
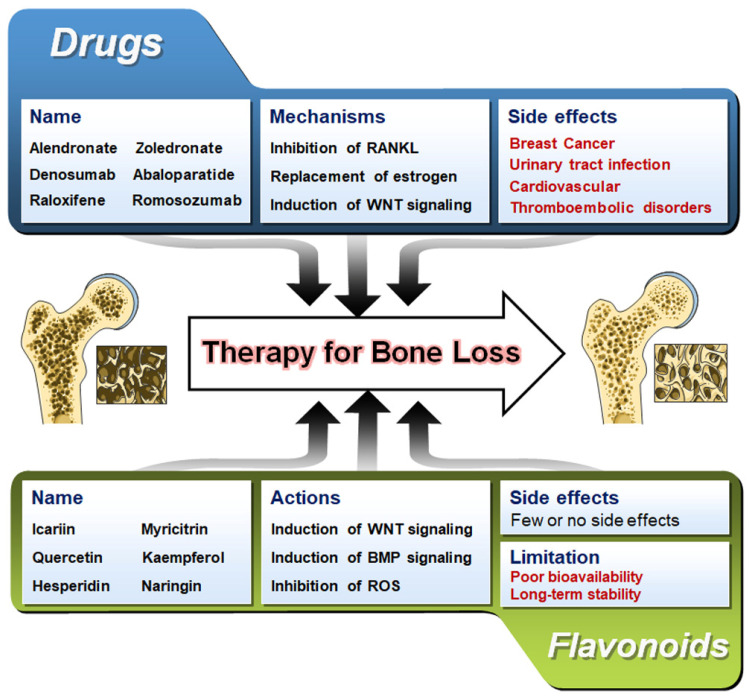
A comparison of flavonoids and available pharmaceutical drugs in treating bone loss. Bone images are taken from Servier Medical Art (https://smart.servier.com accessed on 7 February 2023) which is licensed under a Creative Commons Attribution 3.0 Unported License (Suresnes, France).

In this review, we have discussed the role of flavonoids on the various osteogenic signaling pathways and how various studies have highlighted the potential of flavonoids on bone-forming ability. Natural flavonoids are often cost-effective and possess fewer side effects than their chemically synthesized counterparts (Figure 6). Though tons of research has been performed to decipher the mechanism of action of flavonoids on osteogenesis and osteoclastogenesis, no flavonoids have been approved as a drug for any bone diseases. However, in recent times, to validate the effect of flavonoids, few studies have been undertaken to assess their potential in human subjects (Table 4). Moreover, a few patents have been registered for flavonoids’ role in treating bone-related diseases (Table 5). More such clinical trials are required to tap the potential and ability of flavonoids to influence the bone remodeling process and project them as potential therapeutics.

Despite the pharmacological potential of flavonoids, dietary flavonoids have a number of drawbacks. When consumed with other food ingredients, flavonoids may experience greater precipitation, complexation, and microbial degradation, significantly affecting their stability and bioavailability [261,262]. Even flavonoids in higher concentrations have the potential to be mutagens and pro-oxidants and can create free radicals and inhibitors of important enzymes involved in the metabolism of hormones. Because of this, flavonoids should only be consumed in reasonable amounts that are generally found in a regular vegetarian diet. High dosages may have more negative effects than positive ones. Increased flavonoid exposure from food or supplementation can potentially overwhelm the body, developing reactive oxygen species and ultimately causing DNA damage. Furthermore, due to the rapid cell proliferation that occurs during fetal development, adverse effects might be amplified and lead to increased sensitivity to phytochemical exposure [263]. When consumed at levels of 1000 mg per day, flavonoids frequently cause nausea, headaches, or tingling in the extremities in some persons. Similarly, a study found that some cancer patients may have liver damage from tea extract supplements. In addition, as the safety of flavonoid supplements in pregnancy and lactation has not been demonstrated, it is better to avoid them during these times [264].

The structural components present in flavonoids are also responsible for their potential side effects. When peroxidases oxidize phenol ring-containing flavonoids, they produce cytotoxic phenoxyl radicals, which co-oxidize the unsaturated lipids moieties such as NADH, GSH, nucleic acids, and ascorbate, generate ROS and induce mitochondrial toxicity [265,266,267,268]. The electrophilic quinone/quinone methides formed by flavonoids with catechol rings have been found to bind to GSH, protein, and DNA [267,269,270,271,272]. Because flavonoids have been demonstrated to both promote [273] and inhibit [274] drug-metabolizing enzymes, there are further reasons to be concerned about mega flavonoid supplementation. If flavonoids and other dietary phenolics are to be employed as therapeutic agents, it is clear that more investigation into their possible side effects is necessary [275].

Thus, a plethora of studies will be required to assess the benefits of the bioactivity of flavonoids and their reported drawbacks as therapeutics. Moreover, research must be continued to develop materials or delivery methods that can deliver controlled release kinetics and degradation and directly influence the rate of new bone formation. While developing such strategies, the researcher has to consider factors such as mimicking the bone microenvironment at the implantation site, promoting angiogenesis, stage of inflammation, and osteogenesis phases of new bone formation. Since flavonoid action is well acclaimed in improving bone health, the efficient delivery methods enabling them with easily absorbable and sustained release would benefit bone regeneration in vivo.

## Figures and Tables

**Table 1:** Flavonoids and their effect on bone.

Class	Example	Source	Function to Bone	Reference
Anthocyanidins	Delphinidin	Grapes, berries, sweet potatoes, and pigmented cabbages	Inhibiting differentiation of osteoclasts	[54,55]
	Cyanidin	Berries, red cabbages, black currant, purple rice bran	Promote osteoblast differentiation	[56,57]
	Malvidin	Red grape skin, blueberries and red wine	Stimulate bone formation	[58,59]
	Petunidin	Chokeberries and Saskatoon berries	Inhibit osteoclastogenesis	[60]
	Peonidin	Raw cranberries	Increase osteoblast differentiation followed by the decrease in osteoclast formation	[61]
Flavanols	Catechin	Red wine, Green tea	Stimulate osteoblast growth	[62]
	EGCG	Green tea	Promote osteogenesis	[63]
	EAF		Reduce osteoclastogenesis	[64]
	ECAP		Antiosteoclastogenic activity	[65]
Flavanones	Hesperetin	Citrus fruit	Inhibit osteoclast formation	[66]
	Hesperidin		Promote osteogenesis	[67]
	Naringenin	Grape fruit, tomatoes	Inhibit osteoclastogenesis	[68,69]
	Eriodictyol	Citrus fruit	Inhibit osteoclastogenesis	[70]
Flavanonols	Taxifolin (Dihydroquercetin)	Green tea	Promote osteogenic differentiation	[71]
	Astilbin	Wine, plants	Inhibit osteoclastogenesis	[72]
Flavones	Luteolin	Celery, Cabbage, honeysuckle	Promote osteogenic differentiation	[73]
	Tangeretin	Orange peels	Inhibit osteoclast formation	[74]
	Corylin	Psoralea Fructus	Induce osteoblastogenesis	[75]
	Apigenin	Olive, parsley and apple	Inhibit formation of osteoclast	[76]
	Chrysin	Mushroom, chamomile, honey	Enhance osteogenesis	[77]
	Nobiletin	Citrus fruit	Enhance osteoblastogenesis	[78]
	Baicalein	Scutellaria baicalensis root chinese herb	Stimulate differentiation of osteoblast	[79]
Flavonols	Quercetin	Onions, broccoli, grapes, berries and red wine	Promote osteogenic differentiation Inhibit osteoclast activation	[80,81]
	Kaempferol	Green leafy vegetables	Induce osteogenic activity	[20]
	Galangin	Lesser galangal	Inhibit osteoclastogenesis	[82]
	icariin	Horny goat weed	Induce osteoblast differentiation	[83,84]
	Rutin	Buckwheat	Promote osteoblast differentiation	[85]
	Myricetin	Berries, nuts, red wine	Enhance osteoblast differentiation	[86]
	Fisetin	Apples, grapes and strawberries	Promote osteoblast differentiation	[5]
	Isorhamnetin	Pears, olive oil, tomato sauce and wine	Inhibit osteoclastogenesis	[87]
Isoflavones	Genistein	Soy-based foods	Promote osteoblastogenesis	[21,88]
	Daidzein	Soybeans, tofu	Promote osteoblast proliferation and differentiation	[89]
	Glycitein	Soycheese, soymilk	Decrease osteoclast formation	[90]
	Puerarin	Root of Pueraria	Accelerate osteoblast differentiation	[91]
	Equol	Soybeans	Promote osteoblast proliferation and differentiation	[92]
	Cladrin	Soybeans	Stimulate osteoblast differentiation	[93]
	Calycosin	Soybeans, peanuts	Inhibit osteoclastogenesis	[94]
	Formononetin	Beans, soy	Suppress osteoclastogenesis	[95]

**Table 2:** Therapeutical options for the treatment of bone loss and their mechanisms of action and side effects. (Protelos^®^ and Osseor^®^ (Servier, France)).

Anti-Resorptive Agents			
Drug Class/Name	Actions	Side Effects	References
Nitrogen-containing bisphosphonates Alendronate Ibandronate Risedronate Zoledronate	Restraint of the mevalonate pathway through disruption of protein prenylation by suppressing the farnesyl pyrophosphate synthase enzyme Release of osteoclasts	Dysphagia Nausea/Flatulence Gastritis Constipation/Diarrhea	[208,211,218,219]
Non-nitrogen-containing bisphosphonate Clodronate Etidronate Tiludronate	Disturbance the cell metabolism by using their metabolites instead of ATP Osteoclast apoptosis	Acid regurgitation Esophageal ulcers Hypocalcemia Osteonecrosis of the jaw Atypical femoral fracture	[213,220,221,222]
Monoclonal antibody against RANKL Denosumab	Inhibition of RANK/RANKL signaling pathway through competitive binding to RANKL Inhibition of differentiation and function of osteoclasts	Gastrointestinal disorders Musculoskeletal-related pain Osteonecrosis of the jaw Atypical femoral fracture	[218,223,224]
Selective estrogen receptor modulator Bazedoxifene Lasofoxifene Raloxifene Tamoxifen	Combine with estrogen receptors and acting selective estrogenic activity Osteoclast apoptosis	Cramps of muscle Venous thromboembolic disorder Stroke	[218,225,226]
Estrogen replacement therapy Oestrogen	Induction of caspase-8 cleavage through the combination of Fas and Fas ligand on the surface of pre-osteoclasts after promoting the transcription of Fas ligand by binding to estrogen receptor-α Osteoclast apoptosis	Breast cancer Heart disease Stroke Venous thromboembolic disorder	[208,216,220]
Calcitonin	Reducing the level of blood calcium by binding to calcitonin receptors on osteoclasts Transcriptional regulation through cyclic adenosine monophosphate/protein kinase A-cAMP-response element binding protein pathway	Nasal adverse reaction Anti-calcitonin antibody formation Hypocalcemia Prostate cancer	[227,228,229]
Cathepsin K inhibitor Balicatib Odanacatib ONO-5334	Preventing the collagen cleaves by binding to cathepsin K Increase bone mineral density by suppressing the osteoclast activity by inhibiting the cathepsin K	Stroke Pycnodysostosis Atypical femoral fracture	[230,231,232]
Strontium ranelate Protelos^®^ Osseor^®^	Reduction of osteoclast activity by inducing the production of osteoprotegerin Induction of osteoclast apoptosis through directly binding to the calcium sensing receptors	Venous thromboembolic disorder Myocardial infarction Cardiovascular disorder	[231,233,234]
**Anabolic Agents**			
**Drug Class/Name**	**Actions**	**Side Effects**	**References**
Parathyroid hormone analogue Teriparatide	Increasing bone formation through binding to the parathyroid hormone-1 receptor on osteoblasts	Nausea Headache/Dizziness Leg cramps Osteosarcoma	[235,236,237]
Parathyroid hormone related-protein Abaloparatide	Increasing bone formation through binding to the parathyroid hormone-1 receptor on osteoblasts	Injection site reaction Dizziness Myalgia Gastrointestinal symptoms Osteosarcoma	[238,239,240]
Monoclonal antibody against sclerostin Romosozumab Blosozumab	Increase the activation of Wnt signaling by degrading the sclerostin	Stroke Myocardial infarction Cardiovascular disorder	[241,242,243]

**Table 3:** Advantages and Disadvantages of delivery systems for flavonoids.

Types	Advantages	Disadvantages	References
Lipid nanoparticles	Low toxicity Low side effects High biocompatibility	High production costs Comparatively reduced loading efficiency Expulsion	[182,252]
Liposome	Low toxicity Low side effects Increased efficacy High biocompatibility	High production costs Short half-life Expulsion Low solubility Low stability	[185,253]
Metal nanoparticles	High stability Possibility of large-scale production High biocompatibility	Toxicity Complicated synthetic route	[186,254]
Bioactive glass nanoparticles	High biocompatibility	Mechanical weakness Low fracture resistance	[187,255]
Micelles	Easy and reproducible scale-up Low side effects Longer circulation	Low stability	[189,256]
Scaffolds	High biocompatibility Low immunogenicity Excellent cytocompatibility	Poor mechanical properties High production costs Low reproducibility	[201,257]
HA bioceramic microspheres	Low toxicity High biocompatibility	Poor mechanical properties High production costs Low reproducibility	[203,258]
Phase-transited lysozyme-primed Ti surface	High biocompatibility High biocompatibility Fabricating long-term antibacterial multilayer coatings	Toxicity	[204,259]
Nano coating	Low toxicity High biocompatibility	Low stability	[205,260]

**Table 4:** Clinical trial studies of flavonoids for treating bone loss.

No.	Flavonoid/Combination	Trial No.	Phase	Status	Disease
1	Calcium, Vitamin D, and Flavonoid supplements	NCT05421819	-	Recruiting	Osteopenia, Postmenopausal osteopenia
2	Hesperidin	NCT00330096	Phase III	Completed	Osteoporosis, Osteopenia
3	Quercetin	NCT05371340	-	Completed	Osteoporosis, Osteopenia
4	Isoflavones	NCT00244907	Phase I	Completed	Osteoporosis, Osteopenia
5	Isoflavone (red clover extract)	NCT02174666	-	Unknown	Postmenopausal osteopenia

**Table 5:** Patents of flavonoids for treating bone loss.

Patent No.	Patent Type	Application No.	Publication Date	Content of the Patent	Inventors	Applicant
US2008003300A1	US	US81952707A	3 January 2008	A combination of flavan and Free-B-Ring flavonoid with Mg/Zn/F-CaP to prevent osteoporosis and other bone diseases.	Gaffar Maria C.	-
KR100345825B1	Republic of Korea	KR20000003048A	24 July 2002	Method to isolate, identify and extract flavonoids and serotonins lignans for enhancing bone formation.	Choi Sang-Won; Wonjeong Lee; Kang Ga-hwa; Seonghee Cho	Woori Honghwa Farming Association Corporation
CN102600126B	China	CN 201210071686	4 May 2011	The implication of prenylated flavonoid for the prevention of osteoporosis and the accelerates the process of bone formation	Li Rongtao; Li Yanping; Deng Xuliang; Li Hongmei	Kunming University of Science and Technology
WO2002017909A1	WIPO (PCT)	PCT/KR2001/000368	7 March 2003	The employment of quercetin against osteoporosis	Chung-Sook Kim; Hye-Kyung Ha; Kye-Yong Song	Korea Institute of Oriental Medicine
CN103989732A	China	CN201410183257.5A	11 July 2017	The employment of kaempferitrin, total flavonoids and Beggarweed extract against osteoporosis	Zheng Chengjian; Qin Luping; Ma Xueqin; Zhang Qiaoyan; Han Ting; Zhang Hong	Second Military Medical University
